# High SMAD7 and p-SMAD2,3 expression is associated with environmental enteropathy in children

**DOI:** 10.1371/journal.pntd.0006224

**Published:** 2018-02-07

**Authors:** Sana Syed, Vincenzo Dinallo, Najeeha T. Iqbal, Laura Di Iorio, Davide Di Fusco, Shan Guleria, Beatrice C. Amadi, Kamran Sadiq, Christopher Moskaluk, S. Asad Ali, Paul Kelly, Giovanni Monteleone

**Affiliations:** 1 Department of Paediatrics and Child Health, Aga Khan University, Karachi, Pakistan; 2 Department of Pediatrics, Division of Gastroenterology, Hepatology, & Nutrition, University of Virginia, Charlottesville, United States of America; 3 Department of Systems Medicine, University of Rome 'Tor Vergata', Rome, Italy; 4 Tropical Gastroenterology and Nutrition group, University of Zambia School of Medicine, Lusaka, Zambia; 5 Blizard Institute, Barts and The London School of Medicine, Queen Mary University of London, London, United Kingdom; University of California San Diego School of Medicine, UNITED STATES

## Abstract

Enteropathies such as Crohn’s disease are associated with enteric inflammation characterized by impaired TGF-β signaling, decreased expression of phosphorylated (p)-SMAD2,3 and increased expression of SMAD7 (an inhibitor of SMAD3 phosphorylation). Environmental enteropathy (EE) is an acquired inflammatory disease of the small intestine (SI), which is associated with linear growth disruption, cognitive deficits, and reduced oral vaccine responsiveness in children <5 y in resource-poor countries. We aimed to characterize EE inflammatory pathways by determining SMAD7 and p-SMAD2,3 levels (using Western blotting) in EE duodenal biopsies (N = 19 children, 7 from Pakistan, 12 from Zambia) and comparing these with healthy controls (Ctl) and celiac disease (CD) patients from Italy. Densitometric analysis of immunoblots showed that EE SI biopsies expressed higher levels of both SMAD7 (mean±SD in arbitrary units [a.u.], Ctl = 0.47±0.20 a.u., EE = 1.13±0.25 a.u., p-value = 0.03) and p-SMAD2,3 (mean±SD, Ctl = 0.38±0.14 a.u., EE = 0.60±0.10 a.u., p-value = 0.03). Immunohistochemistry showed that, in EE, SMAD7 is expressed in both the epithelium and in mononuclear cells of the lamina propria (LP). In contrast, p-SMAD3 in EE is expressed much more prominently in epithelial cells than in the LP. The high SMAD7 immunoreactivity and lack of p-SMAD3 expression in the LP suggests defective TGF-β signaling in the LP in EE similar to a previously reported SMAD7-mediated inflammatory pathway in refractory CD and Crohn’s disease. However, Western blot densitometry showed elevated p-SMAD2,3 levels in EE, possibly suggesting a different inflammatory pathway than Crohn’s disease but more likely reflecting cumulative protein expression from across all compartments of the mucosa as opposed to the LP alone. Further studies are needed to substantiate these preliminary results and to illustrate the relationship between SMAD proteins, TGF-β signaling, and inflammatory cytokine production, all of which may be potential therapeutic targets.

## Author summary

Environmental enteropathy (EE) is a disease found in children living in low- and middle-income countries, involving gut inflammation similar to that seen in Crohn’s disease or celiac disease. The inflammation leads to poor absorption of nutrients that these children would normally get in their diets, and this malabsorption in turn leads to poor growth, problems with cognitive development, and reduced efficacy of orally-administered vaccines. However, very little is known about what biochemical pathways lead to the inflammation characteristic of EE. One possible pathway involves proteins called TGF-β, p-SMAD2,3, and SMAD7. Normally, TGF-β stops inflammation with help from p-SMAD2,3; on the other hand, SMAD7 blocks this process, causing increased inflammation. Previous research on Crohn’s disease has shown that drugs like Mongersen, which reduces the expression of SMAD7, can help treat the inflammation. We therefore aimed to see if SMAD7 played a similar role in EE. We found that SMAD7 levels were elevated in EE just like in Crohn’s disease, but we found that p-SMAD2,3 levels were also higher in EE than healthy patients, which was surprising given its anti-inflammatory effects. In order to find a treatment for EE, more work is needed to determine the exact role of these SMAD proteins in EE.

## Introduction

Environmental enteropathy (EE) is an acquired inflammatory disease of the small intestine, characterized histologically by blunted villi, elongated crypts, and increased lymphocytic infiltration of the lamina propria (LP) [1–3]. These structural and inflammatory changes characteristic of EE are tightly linked to the malabsorption characteristic of EE. Subsequent malnutrition is associated with linear growth faltering (e.g. stunting, with a length-for-age z score [LAZ] less than 2) in children affected with EE, and the chronic nature of the disease means that stunting is likely to lead to long-term impaired cognitive development [4, 5]. In addition, poor responses to nutritional interventions [6] and especially reduced immunogenicity after oral vaccination [7, 8] have been linked to EE. Given that diarrheal disease is the second leading cause of death among children under 5 years of age [9] and that undernutrition was responsible for 45% of all child deaths in 2011 [10], it is clear that the effects of EE on nutrient absorption and immunizations carries with it a substantial mortality risk. In short, EE stands to undermine public health efforts in low- and middle-income countries, yet relatively little is known about its pathogenesis. One hypothesized mechanism involves an increase in the permeability of the gut mucosa, allowing microflora into compartments from which they would normally be absent; this translocation leads to further inflammation, which increases the disturbance of normal mucosal structure and function in a vicious cycle [11, 12]. Even in light of this hypothesis, the specific biochemical pathway by which EE results in inflammation is unknown.

**Fig 1:**
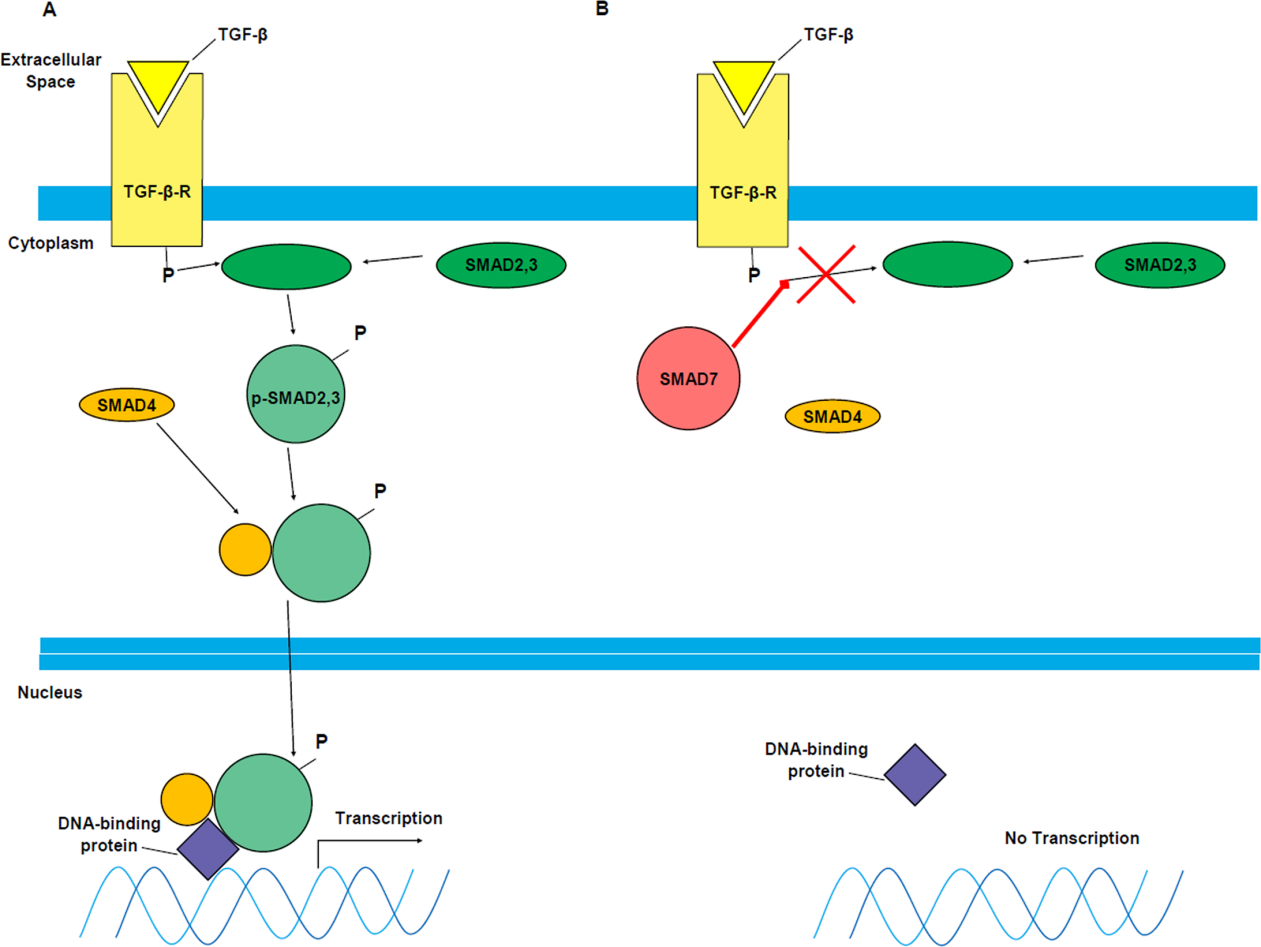
Hypothesized simplified role of SMAD proteins in TGF-β signaling in inflammatory enteropathies. A) Activated SMAD proteins are shown as circles while inactivated SMAD proteins are shown as ovals. TGF-β binds to the TGF-β receptor (TGF-β-R), and this activated receptor then phosphorylates SMAD2,3 into p-SMAD2,3. p-SMAD2,3 forms a heterodimer with SMAD4, and this whole complex moves to the nucleus. There the complex binds to a DNA-binding protein, allowing for the transcription of immunosuppressive genes (e.g. cyclin-dependent kinase inhibitors that antagonize cell proliferation). B) SMAD7 inhibits the phosphorylation of SMAD2,3 by the activated TGF-β-R. This prevents the transcription factor complex from forming. Without this complex, immunosuppressive genes are not transcribed. Effectively, SMAD7 inhibits the anti-inflammatory activity of TGF-β, explaining why high SMAD7 levels are associated with IBD-associated inflammation. Mongersen is an anti-SMAD7 oligonucleotide that inhibits the expression of SMAD7 by targeting its mRNA transcript for degradation [21]. Oral administration of Mongersen therefore blocks SMAD7 activity, restoring TGF-β signaling (yellow box). Figure derived from Heldin, 1997 [15].

Transforming growth factor-β (TGF-β) is an immunosuppressive cytokine important for oral antigen tolerance, suppressing Th1 and Th2 cell responses, and controlling inflammation in the gut [13, 14]. This anti-inflammatory function in the gut is mediated by intracellular SMAD proteins via a simplified hypothesized pathway outlined in Fig 1 [15–17]. TGF-β binds to a receptor on the cell surface, leading to the phosphorylation of SMAD2,3. These proteins then heterodimerize with SMAD4 and travel to the nucleus where they complex with a DNA-binding protein and act as a transcription factor for anti-inflammatory genes (Fig 1A). However, SMAD7 is an inhibitor of the TGF-β-mediated phosphorylation of SMAD2,3 and effectively blocks the immunosuppressive functions of TGF-β (Fig 1B).

Other enteropathies have been linked to this TGF-β-SMAD pathway, highlighting its potential role in EE. In refractory celiac disease (CD), which is characterized by the persistence of malabsorption and villous atrophy despite a gluten-free diet and in the absence of other disorders, high SMAD7 levels were associated with defective TGF-β signaling and elevated production of inflammatory cytokines [18]. Not only has inflammatory bowel disease (IBD) been associated with high SMAD7 levels [19], but studies have also asserted that knocking out SMAD7 in animals and pharmacologically blocking it in clinical studies can lead to reduced gut inflammation and improvement in symptoms [20, 21]. In light of these observations and in order to determine if SMAD7 is a viable target for EE therapy, our aim in the present study was to characterize TGF-β and SMAD protein expression in EE patients, comparing these data to expression levels in both healthy controls and patients with CD. We hypothesized that, as in refractory CD and IBD, EE would be associated with elevated SMAD7 expression, suggesting that defective TGF-β signaling is the source of inflammation in EE.

## Materials and methods

### Patients and samples

Children in this analysis included 43 patients, of whom 19 had EE and 24 were Italian children who either had celiac disease or were regarded as healthy controls (Table 1).

**Table 1:** Characteristics of biopsy study subjects from Zambia and Pakistan.

	Overall (N = 19)	Zambia (N = 12)	Pakistan (N = 7)	
Baseline Characteristics	Mean ± SD or N (%)	Mean ± SD or N (%)	Mean ± SD or N (%)	p-values^ψ^
Female	9 (47.4%)	5 (41.7%)	4 (57.1%)	0.65
Low birth weight, <2.5 kg	4 (22.2%)	2 (18.2%)	2 (28.6%)	0.60
Exclusive breastfeeding, months	5 ± 1.4	6 ± 1.4	4 ± 1.0	0.02
**Pre-Biopsy Characteristics**				
Age, years	1.5 ± 0.4	1.3 ± 0.4	1.9 ± 0.1	0.0004
Stunting, LAZ <-2	13 (68.4%)	7 (58.3%)	6 (85.7%)	0.33
Underweight, WAZ <-2	16 (84.2%)	10 (83.3%)	6 (85.7%)	>0.99
Wasted, WLZ <-2	13 (68.4%)	9 (75.0%)	4 (57.1%)	0.62
MUAC, cm	12 ± 1.8	12 ± 1.8	—	—
Diarrhea duration, days^§^	22 ± 40.1	33 ± 49.0	3 ± 1.9	<0.0001
Blood in stools, yes	0 (0%)	0 (0%)	—	—
Presence of edema, no	6 (50.0%)	6 (50.0%)	—	—
Presence of dehydration, yes	2 (16.7%)	2 (16.7%)	—	—
HIV positive	2 (16.7%)	2 (16.7%)	—	—

Notes

^§^Diarrhea duration—in Zambia: maternal recall of number of days the child had diarrhea at the time of presentation/ enrollment into the study, in Pakistan: average monthly episodes of loose stools over 24 months of follow-up

^ψ^p-values were calculated to determine if characteristics between Zambian and Pakistani children were significantly different. Fisher's exact test was used for categorical data, and Mann-Whitney tests were used for comparing two means of continuous data.

Abbreviations—MUAC = mid upper arm circumference; HIV = human immunodeficiency virus; LAZ = length-for-age *z* score; WAZ = weight-for-age *z* score; WLZ = weight-for-length *z* score

The EE subjects from Pakistan were part of a larger parent study designed to investigate novel biomarkers of EE [22]. This was a prospective birth cohort (n = 380 children) in the rural district of Matiari, Pakistan, where children were followed monthly for growth faltering. All children received intensive nutritional counseling and those with persistent growth failure (n = 65) received a 12-week, high-calorie nutritional intervention. Children who did not respond to high-calorie nutritional intervention and had clinical concern for malabsorptive organic pathology (with symptoms such as persistent diarrhea, bloating, etc.) were evaluated by a clinical pediatric gastroenterologist, and diagnostic endoscopies were performed on 11 children. These data have been previously reported [22]; in summary one child had celiac disease, and the remainder were considered to have EE. Intestinal tissue from 7 of these children remaining from research and investigative work-up was further stained for evaluation of SMAD-related inflammatory pathways.

The Zambian samples were collected from children in the malnutrition ward of the University Teaching Hospital, Lusaka. This is a clinically active malnutrition unit which treats children with severe acute malnutrition. Children were recruited if they had persistent diarrhea and first line investigations (stool microscopy and culture) had failed to reveal a cause. Children were excluded if they were considered too ill for endoscopy to be carried out safely, or if they had any significant congenital abnormalities. Further details regarding the ward and the children hospitalized there are described by Amadi, et al. [23]. Endoscopy was carried out under anesthesia using ketamine-based protocols.

Endoscopic duodenal biopsies were taken from healthy Italian controls (n = 17) and CD patients (n = 7). Normal controls were subjects presenting to the Department of Systems Medicine at the University of Rome Tor Vergata who underwent upper endoscopy for persistent dyspeptic symptoms and had normal intestinal duodenal mucosa. All CD patient biopsies were from their initial diagnostic endoscopy, at which time they were following a gluten-containing diet and had villous atrophy on histological examination of their duodenal tissue.

### Background characteristics of EE biopsy subjects

Background characteristics of the 19 EE study participants are summarized in Table 1. Our subjects had significant growth faltering with Pakistan (85.7%) having a greater proportion of stunted children than Zambia (58.3%), Zambia (75.0%) having a greater proportion of wasted children than Pakistan (57.1%), and both countries having similar proportions of underweight children. The following clinical characteristics were available only for the Zambian children: mid upper arm circumference [MUAC], presence of blood in the stool, presence of edema, dehydration, and HIV status. Differences in reporting and subject selection meant that the duration of diarrhea was defined differently in the two countries. In Zambia, diarrhea duration was defined as maternal recall of number of days the child had diarrhea at the time of presentation or enrollment into the study (mean duration of 33 days). In Pakistan, diarrhea duration was defined as average monthly episodes of loose stools over 24 months of follow-up (mean duration of 3 days). Certain data were not available for the Pakistani cohort and are therefore not presented in Table 1.

Demographic data for the Italian control and CD patients was not as complete as for the EE patients. The mean±SD age of the 17 control patients was 13±4.3 years while for the 7 CD patients it was 8±3 years. Approximately 76% of control patients were female, as compared to only 43% of celiac patients. These data are summarized in S1 Table.

### Endoscopy and tissue handling

Distal duodenal intestinal mucosa was biopsied endoscopically by a trained pediatric gastroenterologist using either an Olympus Evis Exera III CV-190 pediatric endoscope (Pakistan) or a Pentax EG2490k pediatric endoscope (Zambia). Gastric biopsies were obtained only if the mucosa looked endoscopically abnormal. Endoscopic duodenal biopsies were obtained for all study participants using standard biopsy forceps, oriented prior to fixation (Zambia only), and immediately fixed in 10% neutral buffered formalin and processed for embedding in paraffin for immunohistochemistry (IHC). Paraffin-embedded duodenal biopsies were obtained from Aga Khan University in Pakistan and from the Tropical Gastroenterology and Nutrition Group at the University of Zambia School of Medicine in Lusaka, Zambia. Control subjects were selected from the tissue archives of the University of Rome Tor Vergata in Rome, Italy. Duodenal biopsies from the 24 controls were selected and divided into two distinct groups: healthy normal (n = 17) and CD (n = 7).

### Enteric inflammatory scoring

A tentative but not-yet-validated EE scoring system developed by a consortium of pathologists (S2 Table) was applied to the EE from Zambia and Pakistan. H&E scanned images of biopsies from patients in this study (n = 8 slides from Zambia and n = 7 slides from Pakistan) were made available via a HIPAA-protected online resource from the Washington University in St. Louis as part of the EED biopsy consortium. EE inflammatory scores of patients from Zambia were compared to those from Pakistan using a Mann-Whitney test.

### Western blotting

Duodenal samples were lysed on ice in buffer containing 10 mM HEPES [pH 7.9], 10 mM KCl, 0.1 mM EthyleneDiamineTetraacetic Acid (EDTA), 0.2 mM Ethylene Glycol-bis (β-aminoethyl ether)-N,N,N',N'-Tetraacetic Acid (EGTA), and 0.5% Nonidet P40 supplemented with 1 mM dithiothreitol (DTT), 10 mg/ml aprotinin, 10 mg/ml leupeptin, 1 mM phenylmethylsulfonyl fluoride (PMSF), 1 mM Na_3_VO_4_, and 1 mM NaF. Lysates were clarified by centrifugation and separated on sodium dodecyl sulphate (SDS)-polyacrylamide gel electrophoresis. Blots were incubated with antibodies against SMAD7 (1 μg/ml MAB2029, R&D Systems, Minneapolis, MN, USA), total ERK (sc-94, Santa Cruz Biotechnology, Santa Cruz, CA, USA), p-SMAD2,3 (#8828, Cell Signaling Technology, Danvers, MA, USA), total and phosphorylated STAT4 proteins (sc-486, sc-28296, Santa Cruz Biotechnology). Densitometry data for SMAD 7 and SMAD2/3 was normalized using ERK1/2 by calculating values expressed as arbitrary units (a.u.) using ERK1/2 as the denominator. Multiple Western blots were performed (blots were stripped and then incubated with different antibodies), and densitometry data from each Western blot were taken individually for analysis given inherent variability in densitometry as is established [24]. Limited availability of tissue samples made it impractical to perform transcriptional-level analyses using RNA.

### Enzyme-linked immunosorbent assay (ELISA)

Total proteins extracted from duodenal biopsy samples as described above were analysed for the content of active TGF-β1 using a specific ELISA kit (R&D Systems) in accordance with the manufacturer’s instructions. TGF-β1 was assessed in Pakistan samples but not in those from Zambia, as the mucosal material received from the latter group was too small, and the amount of proteins extracted from such samples was not sufficient to perform an ELISA.

### Histopathological analysis and immunohistochemistry

Paraffin-embedded sections of biopsy samples were deparaffinized, dehydrated through xylene and ethanol and incubated with rabbit anti-SMAD7 (sc-11392, Santa Cruz Bitechnology), anti-p-SMAD3 (ab52903, Abcam, Cambridge, UK) or isotype control antibodies (R&D Systems). Immunoreactive cells were visualized using MACH4 Universal HRP-Polymer kit (Biocare Medical, Concord, CA, USA) with 3,3'-Diaminobenzidine (DAB) (Dako North America, Carpinteria, CA, USA) as a chromogen system, according to the manufacturer's instructions, and lightly counterstained with hematoxylin. Isotype control sections were prepared under identical immunohistochemical conditions, replacing the primary antibody with a purified control isotype.

### Data management and statistical analysis of intestinal tissue

WHO Child Growth Standards (WHO Anthro, Geneva, Switzerland) were used to calculate z-scores, and categorized stunting as a height-for-age z-score < -2 SD (standard deviation), underweight as a weight-for-age z-score < -2 SD and wasting as a weight-for-height z-score < -2 SD. Differences in protein expression between EE, CD, and control groups were compared using the Mann-Whitney U non-parametric test for comparing two groups and the Kruskal-Wallis non-parametric test for comparing more than two groups. Statistical analysis and graphical representation of the data were executed using the GraphPad Prism version 6.00 for Windows and GraphPad Software (La Jolla, California, USA, www.graphpad.com). A p-value <0.05 was considered statistically significant.

### Ethics statement

Institutional approval was granted by the Aga Khan University Ethical Review Committee (ERC number 2446), University of Zambia Biomedical Research Ethics (ref006-01-13, approved April 11, 2013), and the University of Rome Tor Vergata (ref. 0003090/2016). In all cases, written consent was obtained from parents/caregivers after a full explanation of the purpose of the procedure, its risks and benefits. Data from this study are not available in a public repository but can be made available upon request.

## Results

### TGF-β and p-STAT4 expression levels

**Fig 2:**
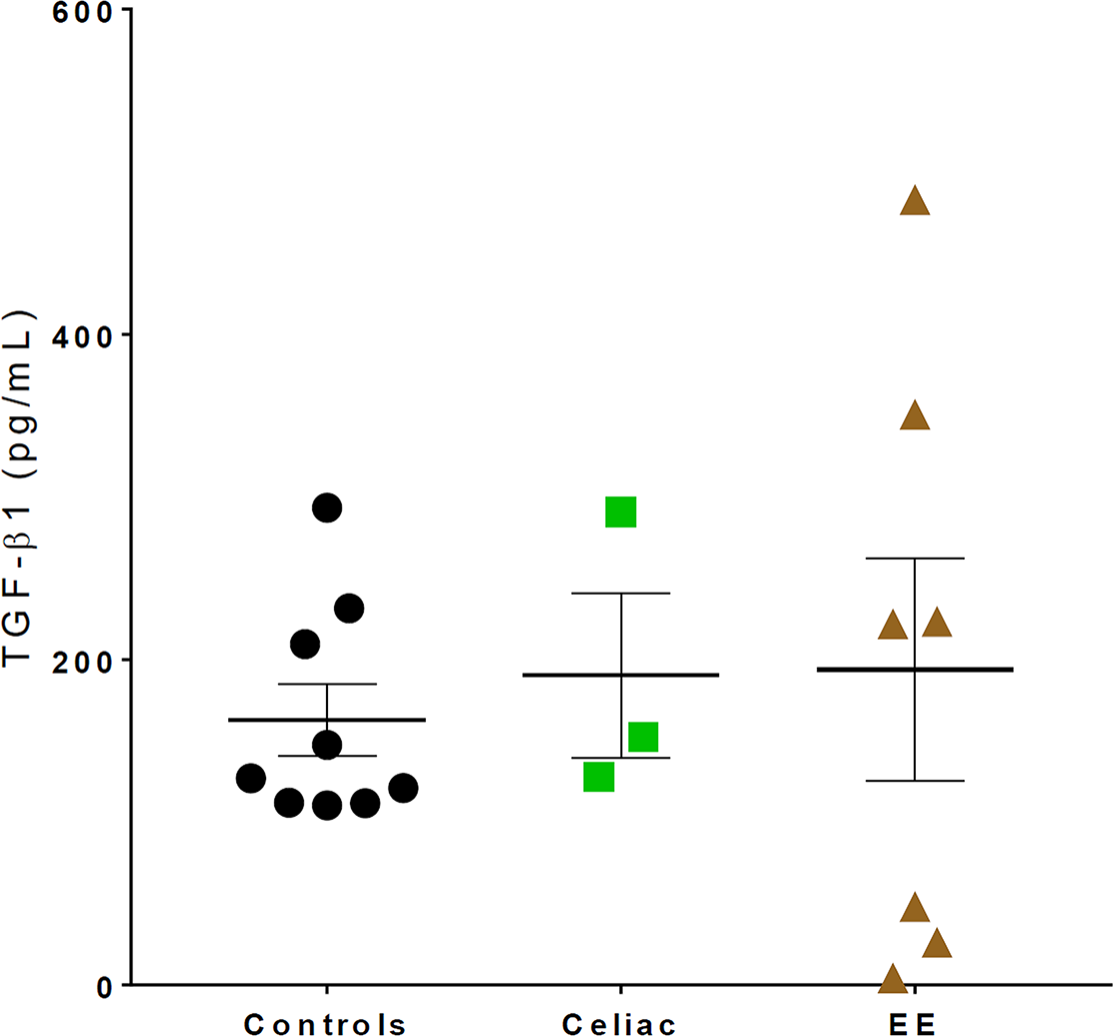
TGF-β expression data. TGF-β expression levels were not significantly different in healthy controls, CD patients, and EE patients (Ctl mean±SD = 162.7±66.3 pg/mL, CD = 190.4±87.8 pg/mL, EE = 193.9±180.4 pg/mL, p-value = 0.793).

Expression of TGF-β in the biopsy samples from EE patients, CD patients, and controls is shown in Fig 2. There were no significant differences in the production of TGF-β between the three groups (p-value = 0.793). While p-STAT4 was expressed in EE patients, CD patients, and healthy controls, it was not expressed at significantly different levels in the three groups (p-value = 0.1114) (S1 Fig).

### SMAD7 and p-SMAD2,3 expression levels in EE subjects

**Fig 3:**
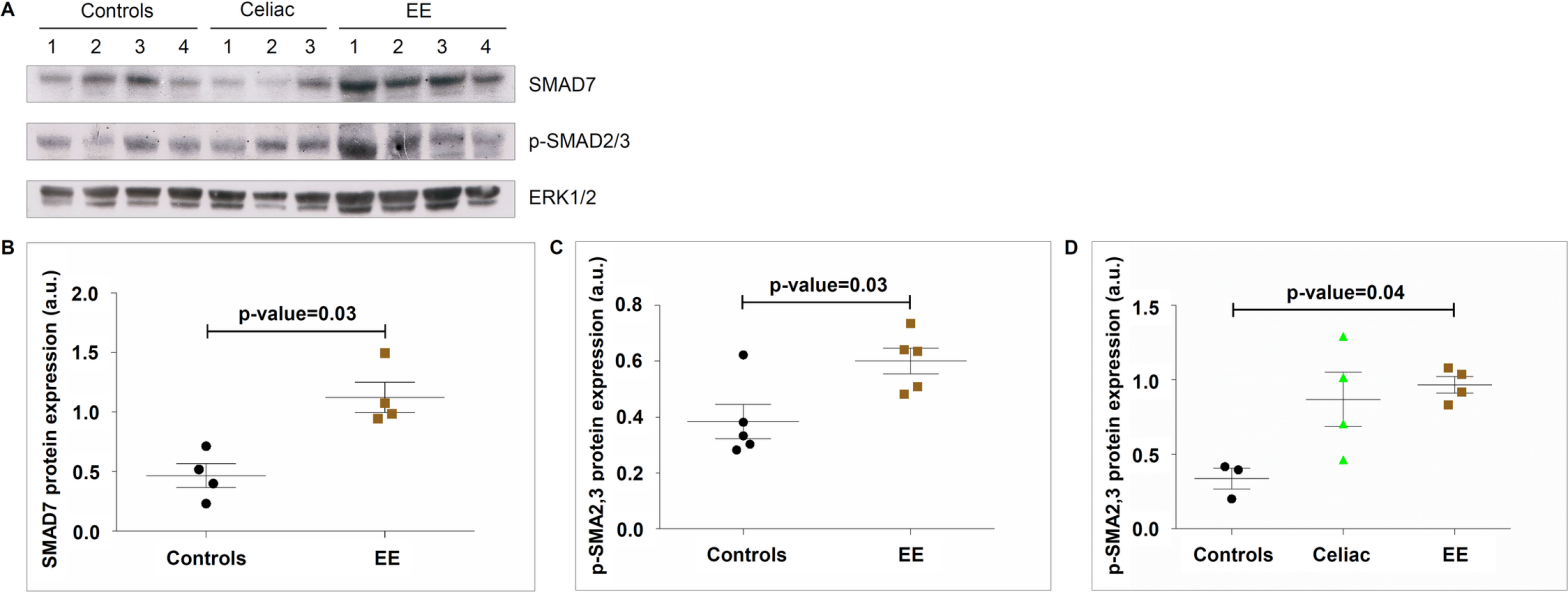
SMAD protein expression data from healthy controls, celiac disease, and environmental enteropathy. A) Representative western blot example investigating SMAD7; p-SMAD2,3 expression (using ERK1/2 as a normalizing protein) from controls (Ctl), celiac disease (CD), and environmental enteropathy (EE) patients. Note that for p-SMAD2,3, patients 3 and 4 have a double band that controls lacked. This band was likely an artifact and consequently was not considered in the densitometric analyses. B) Elevated SMAD7 densitometry levels in EE compared to controls (Ctl mean±SD = 0.47±0.20 a.u., EE = 1.13±0.25 a.u., p-value<0.05). Corresponds to WB5 (see S3 Table). C) Elevated p-SMAD2,3 densitometry levels in EE compared to controls (Ctl mean±SD = 0.38±0.14 a.u., EE = 0.60±0.10 a.u., p-value<0.05). Corresponds to WB2 (see S3 Table). D) Elevated p-SMAD2,3 densitometry levels in EE and CD compared to controls, with significant differences only between EE and controls (p-value<0.05) (Ctl mean±SD = 0.34±0.12 a.u., CD = 0.87±0.36 a.u., EE = 0.97±0.11 a.u.). Corresponds to WB1 (see S3 Table).

An example of the Western blots used to analyze SMAD protein expression can be seen in Fig 3A. Some of the Western blots included only healthy controls and EE patients in the comparisons (Fig 3B and 3C) while others compared controls, CD patients, and EE patients (Fig 3D). Densitometric analysis of these immunoblots showed that duodenal biopsies from EE patients expressed SMAD7 more highly (mean±SD level of 1.13±0.25 a.u.) than healthy controls (0.47±0.20 a.u., p-value = 0.03;Fig 3B). EE patients expressed p-SMAD2,3 more highly (0.60±0.10 a.u.) than healthy controls (0.38±0.14 a.u., p-value = 0.03; Fig 3C). Densitometric analysis of the Western blots that included samples from all three patient groups demonstrated p-SMAD2,3 expression as follows: controls 0.34±0.12 a.u., CD patients 0.87±0.36 a.u., and EE patients 0.97±0.11 a.u., with controls and EE patients being significantly different (p-value = 0.04) (Fig 3D).

### SMAD7 and p-SMAD3 expression varies depending on location in mucosa

**Fig 4:**
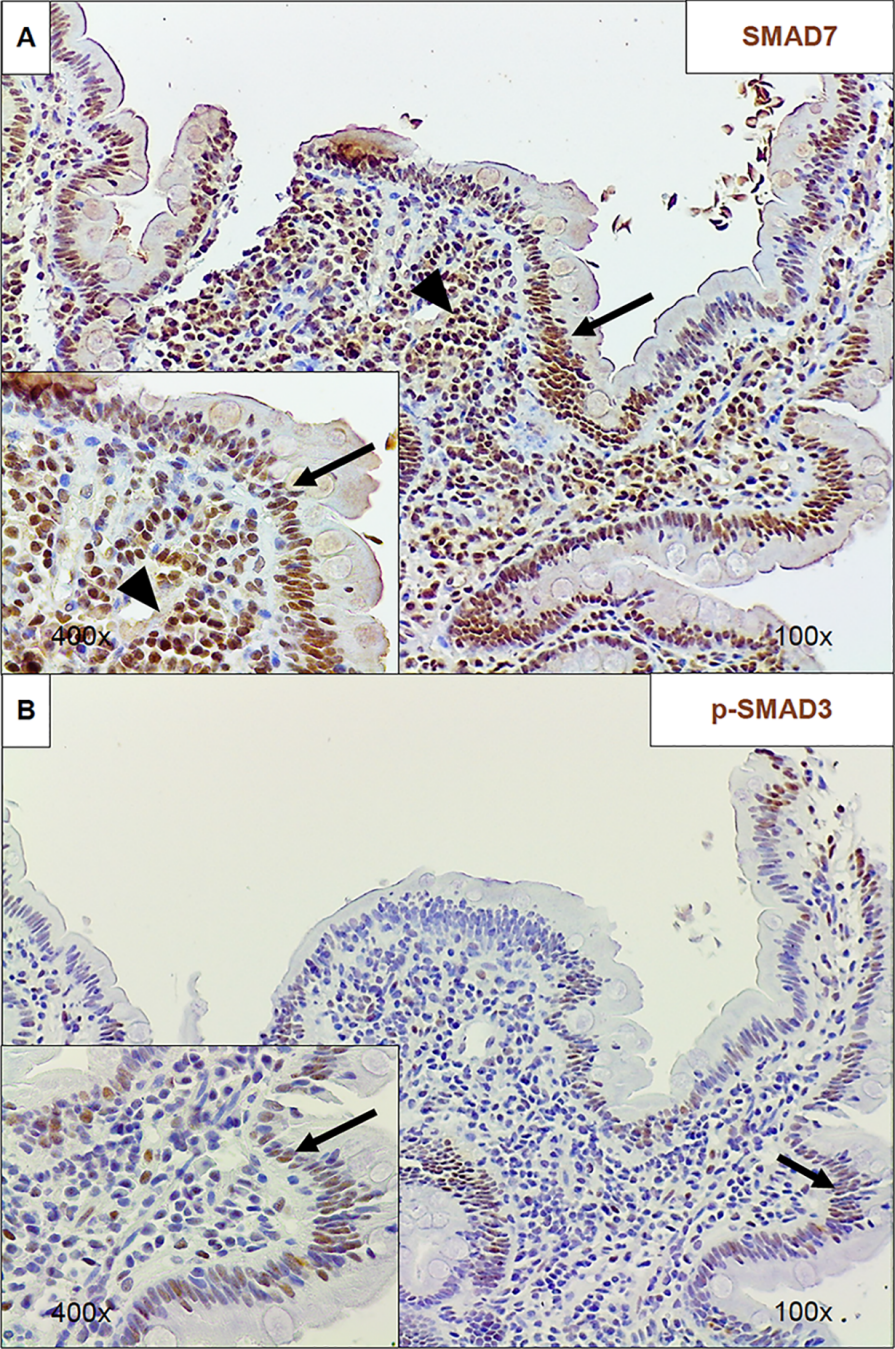
Immunohistochemical staining of duodenal biopsies from EE subjects for SMAD proteins. A) Representative immunohistochemical (IHC) photomicrographs at 100x and 400x from an EE duodenal biopsy showing SMAD7 staining in both the epithelium (arrows) and lamina propria (arrowheads). B) Representative IHC photomicrographs at 100x and 400x from an EE duodenal biopsy showing p-SMAD3 staining in only the epithelium (arrows).

Qualitative immunohistochemistry showed that SMAD7 localized to the nuclei of cells of the epithelium. SMAD7 localization was both nuclear *and* cytoplasmic in mononuclear cells of the lamina propria (LP) (Fig 4A). In contrast, p-SMAD3 was expressed *only* in epithelial cells and not in the LP (Fig 4B).

### Enteric inflammatory scoring

Enteric inflammatory scoring showed that EE patients from Zambia tended to have higher EE inflammatory scores than patients from Pakistan. Mean (SD) of EE scores were as follows: Zambia 13.3(1.8), Pakistan 9.9(3.6), p-value = 0.048 (S2 Fig).

## Discussion

This study explored the role of SMAD proteins and TGF-β in the inflammatory pathway of EE, specifically regarding the expression of SMAD7; p-SMAD2,3; and TGF-β in EE duodenal biopsies from subjects living in Zambia and Pakistan in comparison to Italian CD and control biopsies. Using Western blotting, we determined that both SMAD7 and p-SMAD2,3 were expressed more highly in EE than in controls while CD biopsies only showed an increase in SMAD7 expression compared to controls. Although production of both SMAD7 and p-SMAD2,3 was increased in EE, SMAD7 was expressed both in the duodenal epithelium and the LP while p-SMAD3 was expressed only in the epithelium. Despite these differences between the groups, EE patients, CD patients, and healthy controls did not differ significantly in their duodenal production of TGF-β or p-STAT4 expression.

Our SMAD7 results are consistent with the role of SMAD7 as an inhibitor of TGF-β-mediated enteric anti-inflammatory functions [16, 17]. In mouse models, a lack of TGF-β leads to increased susceptibility to chemical-induced colitis and deficient intestinal mucosal healing [25], and mice with deficient TGF-β signaling produce inappropriate proinflammatory responses to commensal enteric bacteria [26]. Specifically in the cases of inflammatory bowel disease (IBD) [27] and refractory celiac disease (RCD) [18], SMAD7’s role in maintaining intestinal inflammation has been clearly established. In fact, the oral SMAD7 antisense oligonucleotide Mongersen, which blocks SMAD7 functioning, can restore TGF-β signaling and reduce inflammation *in vitro* [19] and in mice with chemical-induced colitis [20]. A phase 2 clinical study later showed Mongersen to be an effective therapy for moderate-to-severe uncomplicated Crohn’s disease [21]. Elevated SMAD7 expression in the context of EE therefore suggests that EE pathogenesis may also rely on SMAD7-mediated inflammation. Additionally, our p-STAT4 data help confirm the presence of Th1-type lamina propria mononuclear cells in EE given that STAT4 is phosphorylated by IL-12, the major Th1-inducing factor in humans, and this transcription factor is expressed only by immune cells [28, 29]. This is in line with the prevalent SMAD7 localization in the same cells and defective TGF-β signaling, as TGF-β is a negative regulator of Th1 cells [13, 14]. Our EE inflammatory score showed that the gross inflammation noted on H&E for subjects from the two countries varied, with Zambian patients tending to show a higher degree of inflammation than Pakistani patients. These findings support our decision to group Zambian and Pakistani EE patients separately in our analyses.

Consistent with what has been shown in refractory CD [18], we demonstrated that TGF-β expression did not differ between EE, CD, and controls. Although we used results obtained by Sedda, et al. to hypothesize that TGF-β expression would not be different between the groups, it should be noted that our celiac controls were newly diagnosed and responsive to a gluten-free diet and therefore did not have refractory celiac disease. Our results seem to suggest that the inflammation in EE is not the result of a lack of TGF-β synthesis and support the role of SMAD7 as an inhibitor of TGF-β signaling. However, such a conclusion would be strengthened by transcriptomic data describing mRNA levels of TGF-β; we plan to analyze these data in our new ongoing study supported by our current results.

Given what is known about the role of SMAD7 and TGF-β in IBD and RCD, our study hypothesis had been that p-SMAD2,3 levels would be decreased in the context of high SMAD7 as SMAD7 inhibits the phosphorylation of SMAD2 and SMAD3 [16, 17]. However, our results indicate that p-SMAD2,3 expression is also elevated in EE compared to controls. These findings seem to suggest that while SMAD proteins and TGF-β may be important in the pathogenesis of EE, the specific pathway may be different than the one responsible for inflammation in IBD and RCD [30, 31]. One possible explanation for this discrepancy involves the compartmentalization of SMAD protein expression in the duodenum. As shown in our qualitative IHC staining, SMAD7 is clearly expressed in both the epithelium and in the LP, and in the LP it seems to be localized to the nucleus and the cytoplasm of mononuclear cells. However, p-SMAD3 expression is essentially absent in the LP. This could suggest that, in the LP specifically, TGF-β signaling may be disrupted, leading to the inflammation present in EE. Because the Western blot densitometric analysis does not differentiate between epithelial and LP expression of SMAD proteins, this LP-specific pathway may have been masked by high expression of both proteins in the epithelium. Future studies should aim to quantify protein expression in the two layers of the duodenal mucosa in order to clarify the relationship between SMAD protein expression and inflammation in the LP and the epithelium.

The goal of this study was to characterize the inflammatory pathway of EE in order to determine which components of that pathway are potential therapeutic targets. Given the success of Mongersen in treating IBD, SMAD7 targeting in EE is an enticing opportunity. However, further research is needed in order to determine the specific role of SMAD7 in EE, especially given that our results possibly suggest that low p-SMAD2,3 levels are not specifically responsible for inflammation in EE. The compartmental localization of SMAD protein expression presents an interesting opportunity for further research on EE inflammation, and Mongersen may still prove useful in treating children with EE.

Our study’s most significant strength was also our largest limitation–namely, safely and ethically obtaining duodenal biopsies in resource limited settings in vulnerable populations. Obtaining these biopsies allowed us to explore how EE changes the tissue of the gut in terms of protein expression and morphology. While we are one of the few groups who have this capacity, we were limited in how many children we could recruit for this study and in how much duodenal biopsy tissue was remaining after clinical diagnostic use for application towards research purposes. This scarcity limited our ability to draw stronger conclusions based on the Western blot and ELISA assays we performed; moreover, the paucity of samples made it challenging to perform additional analyses to further test our hypotheses. For example, we were not able to quantify the differential expression of TGF-β and SMAD proteins in the epithelium versus the LP. Further research is needed to characterize the compartmentalized expression of TGF-β and SMAD proteins as they pertain to EE pathogenesis. We were also unable to explore this pathway at the transcriptional level using RNA and miRNA assays, again due to limited sample availability. Additionally, the children with EE were not age-matched with our Italian controls; future research should aim to use age-matched controls if possible. Despite these limitations, our study demonstrated that SMAD7 expression is increased in EE compared to healthy controls, strengthening the suggestion that therapies targeting SMAD7, such as the Mongersen antisense oligonucleotide, may be viable treatment options in children with EE.

In conclusion, while high SMAD7 duodenal expression is found in EE, the specific pathway by which the inflammation in EE arises is not yet clear. Without disease-modifying treatments for EE, EE will continue to render nutritional and oral vaccination efforts futile. Characterizing the inflammatory pathway of EE is a crucial first step toward developing disease-modifying treatments for EE, and SMAD7 remains a potential target for such treatment.

## Supporting information

S1 Checklist STROBE checklist. (DOC)

S1 Fig p-STAT4 protein expression data from healthy controls, celiac disease, and environmental enteropathy. A) Representative Western blot example investigating p-STAT4 and STAT4 expression (using ERK1/2 as a normalizing protein) from controls (Ctl), celiac disease (CD), and environmental enteropathy (EE) patients. B) Densitometry data showing that expression of p-STAT4 does not differ between healthy controls, CD patients, and EE patients (Ctl mean±SD = 0.55±0.64 a.u., CD = 0.14±0.09 a.u., EE = 0.85±0.31 a.u., p-value = 0.1114).
(TIF)

S2 Fig Environmental enteropathy inflammatory scoring. Enteric inflammatory scores show that patients from Zambia tended to have more inflammation than those from Pakistan, as measured by the criteria laid out in S2 Table. Mean (SD) of the scores were as follows: Zambia 13.3(1.8), Pakistan 9.9(3.6), p-value = 0.048.
(TIF)

S1 Table Characteristics of Italian control and celiac biopsy study subjects. (DOCX)

S2 Table EE scoring system criteria. (DOCX)

S3 Table Setup of the Western blots (WB) used to quantify SMAD7 and p-SMAD2,3 expression. Numbers 1–17 represent healthy controls, 18–24 celiac patients, 25–36 Zambian EE patients, and 37–43 Pakistani EE patients.
(DOCX)
