# Supplementary figures and images for: High SMAD7 and p-SMAD2,3 expression is associated with environmental enteropathy in children
Source: PLoS Negl Trop Dis. 2018 Feb 7;12(2):e0006224. doi: 10.1371/journal.pntd.0006224 (PMC5819826; doi:10.1371/journal.pntd.0006224)

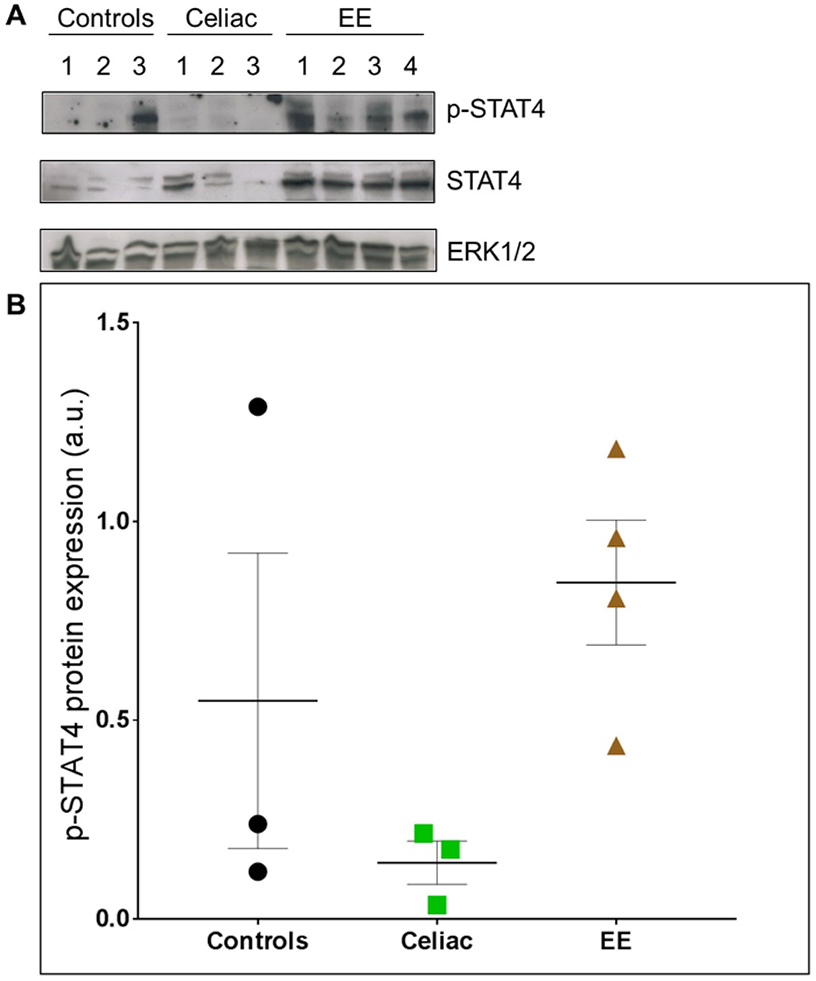

Supplement: S1 Fig — A) Representative Western blot example investigating p-STAT4 and STAT4 expression (using ERK1/2 as a normalizing protein) from controls (Ctl), celiac disease (CD), and environmental enteropathy (EE) patients. B) Densitometry data showing that expression of p-STAT4 does not differ between healthy controls, CD patients, and EE patients (Ctl mean±SD = 0.55±0.64 a.u., CD = 0.14±0.09 a.u., EE = 0.85±0.31 a.u., p-value = 0.1114). (TIF) [file pntd.0006224.s002.tif]

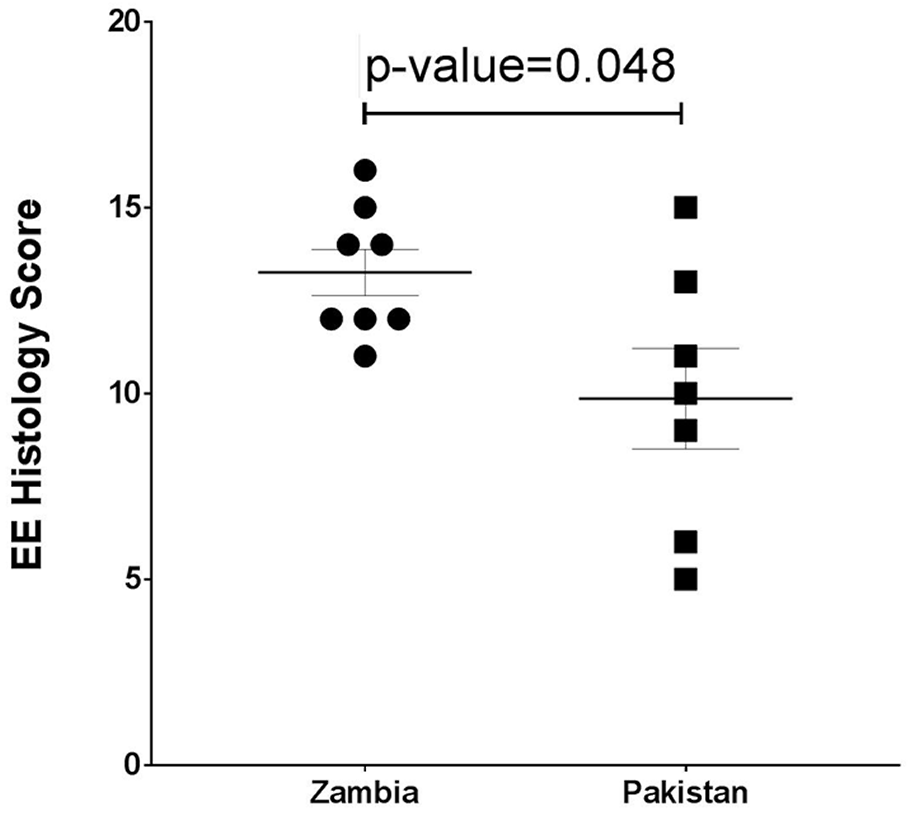

Supplement: S2 Fig — Enteric inflammatory scores show that patients from Zambia tended to have more inflammation than those from Pakistan, as measured by the criteria laid out in S2 Table. Mean (SD) of the scores were as follows: Zambia 13.3(1.8), Pakistan 9.9(3.6), p-value = 0.048. (TIF) [file pntd.0006224.s003.tif]
