# Supplementary material for: High SMAD7 and p-SMAD2,3 expression is associated with environmental enteropathy in children
Source: PLoS Negl Trop Dis. 2018 Feb 7;12(2):e0006224. doi: 10.1371/journal.pntd.0006224 (PMC5819826; doi:10.1371/journal.pntd.0006224)
Supplement: S1 Table — (DOCX) [file pntd.0006224.s004.docx]

| **Table S1** | | | |
| --- | --- | --- | --- |
|  | **Controls (N=17)** | **Celiac (N=7)** |  |
| **Baseline Characteristics** | **Mean ± SD or N (%)** | **Mean ± SD or N (%)** | **p-values^ψ^** |
| Female | 13 (76.5%) | 3 (42.9%) | 0.17 |
| Age (years) | 13 ± 4.3 | 8 ± 3.0 | 0.01 |
| Notes:  ^ψ^p-values were calculated to determine if characteristics between healthy controls and CD patients were significantly different. Fisher's exact test was used for categorical data, and Mann-Whitney tests were used for comparing two means of continuous data. | | | |
