# Supplementary material for: High SMAD7 and p-SMAD2,3 expression is associated with environmental enteropathy in children
Source: PLoS Negl Trop Dis. 2018 Feb 7;12(2):e0006224. doi: 10.1371/journal.pntd.0006224 (PMC5819826; doi:10.1371/journal.pntd.0006224)
Supplement: S2 Table — (DOCX) [file pntd.0006224.s005.docx]

| **Table S2** | | |
| --- | --- | --- |
| **Feature** | **Grade** | **Description** |
| Acute inflammation | 0 | No PMNs observed, or only PMNs in lamina propria with no infiltration of epithelium by PMNs (cryptitis, villitis) |
|  | 1 | 1-2 foci of epithelial PMN infiltration or crypt microabscesses |
|  | 2 | > 2 foci of epithelial PMN infiltration or crypt microabscesses but <50% of mucosa involved |
|  | 3 | > 50% of mucosa involved by epithelial PMN infiltration |
| Chronic inflammation-lamina propria | 0 | No qualitative increase in mononuclear inflammatory cells in lamina propria. Majority of crypt bases are separated by <3 lymphocytes on average. |
|  | 1 | Increase of mononuclear inflammatory cells, based on crypts being separated by 3-5 lymphocytes on average. |
|  | 2 | Increase of mononuclear inflammatory cells, based on crypts being separated by 6-10 lymphocytes on average. |
|  | 3 | Increase of mononuclear inflammatory cells, based on crypts being separated by >10 lymphocytes on average. |
| Intra-epithelial lymphocytes | 0 | No areas observed with epithelial/lymphocyte ratio >20% |
|  | 1 | Lymphocyte/epithelial ratio >20%, but <50%, in less than 50% of mucosa |
|  | 2 | Lymphocyte/epithelial ratio >20%, but <50%, in greater than 50% of mucosa |
|  | 3 | Lymphocyte/epithelial ratio >50% in less than 50% of mucosa |
|  | 4 | Lymphocyte/epithelial ratio >50% in greater than 50% of mucosa |
| Villus architecture | 0 | Majority of villi are >3 crypt lengths long |
|  | 1 | Majority of villi are < 3 crypt lengths long, but > 2 crypt lengths long |
|  | 2 | Majority of villi are < 2 crypt lengths long, but > 1 crypt length long |
|  | 3 | Majority of villi absent, or <1 crypt length long |
| Villus fusion | 0 | None |
|  | 1 | Villus fusion seen in <10% of villi |
|  | 2 | Villus fusion seen in 10-50% of villi |
|  | 3 | Villus fusion seen in >50% of villi |
| Intramucosal Brunner glands | 0 | None observed |
|  | 1 | One or two foci, none involving more than 5 crypt bases |
|  | 2 | More than two foci, or any area of intramucosal Brunner glands involving >5 crypt bases |
| Foveolar cell metaplasia | 0 | Not observed |
|  | 1 | 1-2 villus tips involved |
|  | 2 | > 2 villus tips involved |
| Goblet cell density | 0 | Most villi contain >10 goblet cells |
|  | 1 | Goblet cells <10/ villus, in less than 50% of mucosa |
|  | 2 | Goblet cells <10/ villus, in more than 50% of mucosa |
| Paneth cell density | 0 | >5 Paneth cells/ crypt, on average |
|  | 1 | 1-4 Paneth cells/ crypt, on average |
|  | 2 | <1 Paneth cell/crypt, on average |
| Enterocyte injury | 0 | Majority of enterocytes (90%) show tall columnar morphology |
|  | 1 | Enterocytes show low columnar (<2:1 L:W ratio), cuboidal or flat morphology, in < 50% of mucosa |
|  | 2 | Enterocytes show low columnar (<2:1 L:W ratio), cuboidal or flat morphology, in > 50% of mucosa |
|  | 3 | Any area of mucosal erosion/ulceration |
