# Supplementary material for: High SMAD7 and p-SMAD2,3 expression is associated with environmental enteropathy in children
Source: PLoS Negl Trop Dis. 2018 Feb 7;12(2):e0006224. doi: 10.1371/journal.pntd.0006224 (PMC5819826; doi:10.1371/journal.pntd.0006224)
Supplement: S3 Table — Numbers 1–17 represent healthy controls, 18–24 celiac patients, 25–36 Zambian EE patients, and 37–43 Pakistani EE patients. (DOCX) [file pntd.0006224.s006.docx]

| **Table S3:** | | | | | | | | | |
| --- | --- | --- | --- | --- | --- | --- | --- | --- | --- |
| WB1 | | WB2 | | WB3 | | WB4 | | WB5 | |
| Healthy Controls | 6 | Healthy Controls | 1 | Healthy Controls | 15 | Healthy Controls | 9 | Healthy Controls | 11 |
|  | 7 |  | 2 |  | 16 |  | 10 |  | 12 |
|  | 8 |  | 3 |  | 17 | EE | 35 |  | 13 |
| Celiac Disease | 18 |  | 4 | Celiac Disease | 22 |  | 36 |  | 14 |
|  | 19 |  | 5 |  | 23 |  |  | EE | 40 |
|  | 20 | EE | 29 |  | 24 |  |  |  | 41 |
|  | 21 |  | 30 | EE | 37 |  |  |  | 42 |
| EE | 25 |  | 31 |  | 38 |  |  |  | 43 |
|  | 26 |  | 32 |  | 39 |  |  |  |  |
|  | 27 |  | 33 |  |  |  |  |  |  |
|  | 28 |  | 34 |  |  |  |  |  |  |
